# The effect of childhood sexual abuse on depressive symptoms in female college students: a serial mediation model

**DOI:** 10.3389/fpsyg.2024.1306122

**Published:** 2024-02-13

**Authors:** Haopeng Zheng, Yan Cai, Lei Liu, Biao Peng

**Affiliations:** ^1^College of Marxism, Hunan Normal University, Changsha, China; ^2^School of Marxism, Guangxi University, Nanning, China; ^3^Public Administration School, Guangzhou University, Guangzhou, China; ^4^School of Marxism, Guizhou Medical University, Guiyang, China

**Keywords:** childhood sexual abuse, depressive symptoms, negative core schema, experiential avoidance, female college students

## Abstract

**Objective:**

Childhood sexual abuse (CSA) can have a negative impact on women’s psychological, emotional and social functioning. The purpose of this study was to explore the relationship between CSA and depressive symptoms in female college students, as well as the mediating roles of negative core schema and experiential avoidance.

**Methods:**

515 female college students responded to the Sexual Abuse subscale of the Childhood Trauma Questionnaire, the Depression subscale of the Depression Anxiety Stress Scale, the Brief Core Schema Scales, and the Acceptance and Action Questionnaire – II. The structural equation modeling was used for the mediation analysis.

**Results:**

There was a significant positive correlation between CSA and depressive symptoms in female college students. The theoretical model was well fitted, *χ*^2^/df = 3.422, RMSEA = 0.069, CFI = 0.929, TLI = 0.919. The negative core schema played a mediating role between CSA and depressive symptoms. Experiential avoidance played a mediating role between CSA and depressive symptoms. The negative core schema and experiential avoidance played a serial mediating role between CSA and depressive symptoms.

**Conclusion:**

These results deepen our understanding of the relationship between CSA and depressive symptoms in female college students, and provide theoretical guidance for the prevention of depression in female college students. Attention should be paid to female college students who have experienced CSA, to eliminate the adverse influence of negative core schema on these students. Meanwhile, we should teach female college students to accept themselves as they are, and thereby reduce their use of experiential avoidance strategies.

## Introduction

Depression is a common mental disorder with symptoms including persistent sadness and a lack of interest or pleasure in previously beneficial or enjoyable activities; it can also affect sleep and appetite. Depression is a leading cause of disability worldwide and contributes significantly to the global burden of disease, with an estimated 5% of adults worldwide suffering from the condition (World Health Organization, 2023).

Studies have shown that the incidence, duration, recurrence and comorbidities of depression are higher in women than in men, and this phenomenon is cross-cultural and cross-regional (Salk et al., 2017). Among women, young college students are at high risk for depression. According to the Report on National Mental Health Development in China (2021–2022), women have a higher risk of depression than men, and young people are a high-risk group for depression (Fu and Zhang, 2023). The detection rate of depression risk in the age group of 18 to 24 is as high as 24.1%, which is significantly higher than other age groups (Fu and Zhang, 2023). meta analysis shows that the depressive symptoms detection rate of Chinese college students is as high as 24.71% (Wang et al., 2020). Therefore, the depressive symptoms of female college students deserves attention.

Women are more likely to suffer from depression than men, and the vulnerability of women is closely related to environmental stimuli (Kessler, 2003). In addition to genetic factors, psychosocial factors such as victimization and coping style are specific risk factors for depression in women (Noble, 2005). Women are more likely to be sexually abused than men, and sexual abuse is an important potential risk factor for depression in women (Maniglio, 2010). Children and adolescents who experience CSA are more likely to have symptoms such as depression, anxiety, compulsion, posttraumatic stress symptoms, and sexual problems, which can have lifelong detrimental effects on the development of children and adolescents (Fergusson et al., 2013; Murray et al., 2014). Therefore, it is necessary to explore the mechanisms underlying the link between CSA and depressive symptoms in female college students.

### CSA and depressive symptoms in female college students

CSA, which has been of great concern in the mental health field, is a form of childhood abuse that refers to any sexual act, sexual contact, or sexual exploitation of a child by a guardian (Leeb, 2008). The literature suggests that the global incidence of CSA is between 7.6–7.9% in boys and 18.0–19.7% in girls (Russell et al., 2020). Therefore, compared with men, female childhood sexual abuse needs more attention.

According to the Diathesis-Stress Model, external stress, including both distal stress (e.g., childhood traumatic experiences) and proximal stress (e.g., daily life events), will have a negative impact on mental health (Broerman, 2020). CSA, as early negative stress events experienced by individuals, has been shown in many studies to be a risk factor for mental health problems in adulthood (Hillberg et al., 2011; Hébert et al., 2021). A cross-sectional study showed that CSA is significantly positively correlated with depressive symptoms among female college students (Zhang et al., 2022). Multiple longitudinal studies have shown that CSA is an important predictor of depressive symptoms in female adolescents and adults (Trickett et al., 2011; Girard et al., 2021). In conclusion, CSA, as a negative stressor, is an important factor affecting the depressive symptoms of female college students. Studies have explored the physiological mechanisms underlying the link between CSA and depressive symptoms. Such studies suggest, for example, that CSA leads to female adult depression through long-term effects on hypothalamic–pituitary–adrenal (HPA) axis function (Weiss et al., 1999). Other studies have explored the psychological mechanisms underlying the link of CSA to depressive symptoms: shame or self-blame, avoidant coping strategies (Whiffen and MacIntosh, 2005), interpersonal difficulties (Wilson and Scarpa, 2015), emotion regulation deficits, and stress sensitization (Yaroslavsky et al., 2022) have all been supported as mediating variables between CSA and depressive symptoms. Cognitive schema and emotion regulation strategies are extremely important mediating variables between child maltreatment and depressive symptoms (Li et al., 2020). However, few studies have considered the mediating roles of both negative core schema and experiential avoidance (an important emotion regulation strategy) between CSA and depressive symptoms. At the same time, no studies have considered the sequential mediating effect of negative core schema and experiential avoidance (CSA leads to negative core schema which promote experiential avoidance, which in turn increases risk for depressive symptoms). Therefore, this study aims to explore the mediating roles of negative core schema and experiential avoidance in the influence of CSA on depressive symptoms in female college students respectively, and the sequential mediating role of negative core schema and experiential avoidance in the influence of CSA on depressive symptoms in female college students.

### Mediating role of negative core schema

Beck (1987) developed the concept of schema (or core belief) to refer to one’s most central or core beliefs of oneself, others, and the world, which begins to form in childhood, and which the individual understands to be absolutely true and correct. Beck (1999) divides schemas into three categories: those related to helplessness, those related to unlikability, and those related to worthlessness. Based on Beck’s theory, Young and colleagues divided maladaptive schemas into 18 types (Young et al., 2003). Fowler et al. (2006) divided schemas into positive schemas (both positive self-schema and positive others-schema) and negative schemas (both negative self-schema and negative others-schema). According to the diathesis-stress model, the interaction between external stress and vulnerability has an impact on mental health (Broerman, 2020). Vulnerability includes personality, coping style, and cognitive vulnerability factors (Zou and Yao, 2006). As an important cognitive vulnerability factor, negative core schema is considered to be an important cause of depression and other emotional problems (Dozois and Beck, 2008). Empirical studies have also proven that negative core schema is an important independent risk factor for female depression (Evans et al., 2005), which can lead to symptoms of depression and other mental disorders (Bradley and Mathews, 1983; Bishop et al., 2022).

The formation of negative core schema is closely tied to one’s childhood experience. Childhood trauma not only directly affects the mental health problems of individuals, but also affects the formation of negative core schema. Cognitive behavioral therapy (CBT; Beck, 1991) theory suggests that childhood trauma, as an early stressor, can affect the formation of cognitive vulnerability. CSA experiences are gradually internalized into pathological cognitive structures and maladaptive schema, which are then activated by stressful experiences in adulthood, affecting one’s processing of external information and thus triggering emotional disorders (Beck et al., 1979). The result of a recent meta-analysis also supported a significant correlation between sexual abuse and early onset negative schema (May et al., 2022).

To summarize, CSA experience can affect negative schema, which is an important cause of depressive symptoms. Previous studies have also shown that negative schema are associated with a history of child abuse and adult depression (Cukor and McGinn, 2006); sexual self-schemas play a mediating role between CSA and female sexual dysfunction (Rellini and Meston, 2011); disconnection and rejection schema play mediating roles between childhood trauma (excluding sexual abuse) and depressive symptoms among female college students (Rezaei and Ghazanfari, 2016); cognitive schema plays a mediating role between childhood trauma and high risk of psychosis (Appiah-Kusi et al., 2017). Therefore, negative core schema may play a mediating role between CSA and depressive symptoms in female college students.

### Mediating role of experiential avoidance

Experiential avoidance refers to one’s unwillingness to interact with specific internal experiences (i.e., thoughts, emotions, physical sensations, memories, and behavioral predispositions), or to take steps to change the form, frequency, and context of those experiences (Hayes et al., 1996). According to acceptance and commitment therapy theory (ACT theory), experiential avoidance as an emotional regulation strategy is acquired, rather than being innate, and relational frames play an important role in the formation of experiential avoidance (Hayes et al., 2011). In the context of childhood trauma, individuals tend to form unhelpful relationship frames (i.e., “I am not lovable” as a relationship framing conclusion). The relational frames enable individuals to respond quickly to problems, but they can also cause individuals to rigidly follow rules and form experiential avoidance (Hayes et al., 2011). Previous studies have shown that CSA is a remote factor in triggering maladaptive behaviors such as experiential avoidance. Female college students who have experienced CSA show higher levels of experiential avoidance (Batten et al., 2002). There is a significant positive correlation between CSA and experiential avoidance (Liu et al., 2021).

At the same time, ACT theory suggests that experiential avoidance is an important cause of psychopathology and behavioral problems, and is a common factor in psychological disorders. It is one of the six core concepts of the psychological inflexibility model, and it reinforces psychological inflexibility, which is thought to be the root cause of human suffering and dysfunction (Hayes et al., 2006; Dionne et al., 2013). Empirical research has found that experiential avoidance is not only associated with substance abuse (Kheirabadi et al., 2021), eating disorders (Litwin et al., 2017), self-injury (Angelakis and Gooding, 2021), and other externalizing problems, it has also been shown to be associated with anxiety, compulsion, post-traumatic stress, and depression symptoms (Tull et al., 2004; Orcutt et al., 2020; Akbari et al., 2022), all of which are related to mental health problems (Peng et al., 2021).

In summary, CSA experiences may cause individuals to develop coping styles of experiential avoidance, which can influence depressive symptoms. Empirical research also shows that sexual victimization increases negative adult outcomes through experiential avoidance (Polusny et al., 2004); experiential avoidance plays a mediating role between childhood trauma and problem behaviors (Roche et al., 2019), childhood trauma and depressive symptoms in female college students (Ghazanfari et al., 2018), and childhood trauma and somatic symptoms (Heshmati et al., 2021). Thus, it is reasonable to consider that experiential avoidance may play a mediating role between CSA and depressive symptoms.

### The relationship between negative core schema and experiential avoidance

Both negative core schema and experiential avoidance are important predictors of depressive symptoms, and may play mediating roles between CSA and depressive symptoms. However, few studies have explored the relationship between negative core schema and experiential avoidance. According to the cognitive conceptualization of CBT, early traumatic experience will lead to the formation and development of negative core schema (core beliefs), and these beliefs will affect the development of intermediate beliefs (i.e., attitudes, rules, and assumptions). The intermediate beliefs will affect automatic thinking, and the adoption of adverse coping styles or emotion regulation strategies under negative automatic thinking and emotional distress will result in emotional disorders (Beck, 2011).

Poor coping styles or emotion regulation strategies also strengthen core schema, continuing or intensifying emotional disorders. Experiential avoidance is one particularly maladaptive coping strategy. For example, “I am not lovable” as a core schema will cause negative automatic thinking, shame and even anxiety and depression, and the individual will try to avoid these thoughts and emotions as much as possible, possibly even adopting social avoidance behaviors to deal with them, resulting in the further consolidation of the “I am not lovable” schema and the continuation of the individual’s anxiety and depression (Harris, 2019). Thus, negative core schema may cause individuals to adopt experiential avoidance strategies to deal with their problems, thus maintaining and aggravating symptoms of mental disorders such as depressive symptoms. Empirical studies have shown that experiential avoidance plays a mediating role between disconnection and rejection schemas and depressive symptoms in female college students (Rezaei and Ghazanfari, 2016). As the formation and development of negative core schema is influenced by CSA, negative core schema and experiential avoidance may play a serial mediating role between CSA and depressive symptoms.

### The present study

In previous studies, most of them took childhood trauma as an independent variable, and few studies took CSA as an independent variable, especially few studies explored the mediating roles of negative core schema and experiential avoidance in CSA and depressive symptoms. In addition, we have found no studies examining the serial mediation of negative core schema and experiential avoidance between CSA and depressive symptoms. Therefore, Therefore, we propose the following hypothesis:

*Hypothesis 1* (H1): Negative core schema would play a mediating role between CSA and depressive symptoms in female college students.

*Hypothesis 2* (H2): Experiential avoidance would play a mediating role between CSA and depressive symptoms in female college students.

*Hypothesis 3* (H3): Negative core schema and experiential avoidance would play a serial mediating role between CSA and depressive symptoms in female college students.

## Materials and methods

### Participants and data collection

This was a cross-sectional study, with a sample drawn from two universities in Hunan Province, mainland China, with participants gathered using a convenience sampling method. All the participants were female college students. A total of 600 questionnaires were issued, and after excluding invalid questionnaires such as regular answers and incomplete answers, a total of 515 were recovered, with an effective rate of 85.83%. According to Nunnally and Bernstein (1994) rule of thumb, structural equation modeling (SEM) requires a sample size at least 10 times the number of variables in SEM (which were 310 in our example). Therefore, our sample size (*N* = 515) was sufficient. There were 168 freshmen (32.62%), 181 sophomores (35.15%), 117 juniors (22.72%), and 49 seniors (9.51%). The average age was 20.16 years (SD = 1.21). There were 297 participants (57.31%) from urban areas and 218 participants (42.69%) from rural areas. This study was approved by the Ethics Committee of Hunan Normal University (No. 2023–668), Hunan Province, China. In accordance with the Declaration of Helsinki, informed consent was obtained from all participants prior to them completing the survey, and all were told that their responses were voluntary, anonymous, and confidential.

### Measures

#### CSA

The sexual abuse subscale of the Childhood Trauma Questionnaire, compiled by Bernstein et al. (1997) and revised for Chinese by Zhao et al. (2005), was adopted. The subscale consists of 5 items (e.g., “Someone tried to make me do sexual things or watch sexual things,” Zhao et al., 2005), each of which is rated on a five-point scale (1 = never, 5 = always). A higher score indicates a more severe sexual abuse experience. In this study, the Cronbach α coefficient was 0.900.

#### Depressive symptoms

The Depression Anxiety Stress Scale compiled by Lovibond (1995) was adopted, specifically the Chinese version as revised by Gong et al. (2010). The scale is divided into three dimensions of depression, anxiety and stress, with 7 items in each dimension, for a total of 21 items (e.g., “I feel that life is meaningless,” Gong et al., 2010), each of which is rated on a four-point scale (0 = did not apply to me at all, 3 = applied to me very much, or most of the time) (Lovibond and Lovibond, 1995). The total score is the sum of the scores of each item. In this study, the Cronbach α coefficient of the depression subscale was 0.862.

#### Negative core schema

This study used the Brief Core Schema Scales as compiled by Fowler et al. (2006), specifically the Chinese version which was tested by Chen et al. (2022) and shown to have good reliability and validity. The questionnaire consists of four dimensions: positive-self, positive-others, negative-self, and negative-others, with 6 items in each dimension, and a total of 24 items (e.g., “I am unloved,” Chen et al., 2022). Each item can choose “no” (0 points) and “yes,” choose “yes” also need to choose the degree, from 1 to 4 (1 = somewhat believe, 4 = fully believe). In this study, negative self and negative others were used to reflect the negative core schema. The Cronbach α coefficients of the two sub-dimensions used in this study (i.e., negative-self and negative-others) were 0.838 and 0.894, respectively.

#### Experiential avoidance

The Acceptance and Action Questionnaire - II compiled by Bond et al. (2011) was adopted, specifically the Chinese version translated by Cao et al. (2013). The questionnaire includes 7 items (e.g., “I worry about not being able tocontrol my worries and feelings,” Cao et al., 2013), each of which is rated on a seven-point scale (1 = never, 7 = always). The higher the total score of the questionnaire, the higher the respondent’s level of experiential avoidance. In this study, the Cronbach α coefficient was 0.925.

### Data analysis strategy

All data in this study were managed and analyzed using SPSS 25.0 and Amos 23.0. First, a Harman single factor test was used to test for common method bias (Podsakoff et al., 2003). Exploratory factor analysis obtained 5 factors with feature roots greater than 1 without rotating the axis, and the explanatory variation of the first factor was 27.05%, which is 40% below the critical standard. Therefore, there is no significant common methodological bias in this study. Second, descriptive statistics were conducted on each variable to test their correlation. Third, a bias correction nonparametric percentage bootstrap method was used to test the hypothesized mediating effects.

## Results

### Descriptive statistics

Descriptive analysis and correlation analysis of each variable (see Table 1) showed that the college students’ CSA, negative core schema, experiential avoidance, and depressive symptoms were significantly positively correlated (*p* < 0.01).

**Table 1:** Means, standard deviations, and correlations among variables.

	Mean	SD	1	2	3	4	5
1 CSA	5.503	1.633	1				
2 Negative-self	4.377	3.900	0.292**	1			
3 Negative-others	4.839	3.936	0.243**	0.762**	1		
4 EA	20.753	8.619	0.236**	0.507**	0.417**	1	
5 DS	4.144	3.916	0.298**	0.711**	0.621**	0.592**	1

CSA, childhood sexual abuse; EA, experiential avoidance; DS, depressive symptoms; **p < 0.01.

### Mediating effect test of negative core schema and experiential avoidance

**Figure 1:**
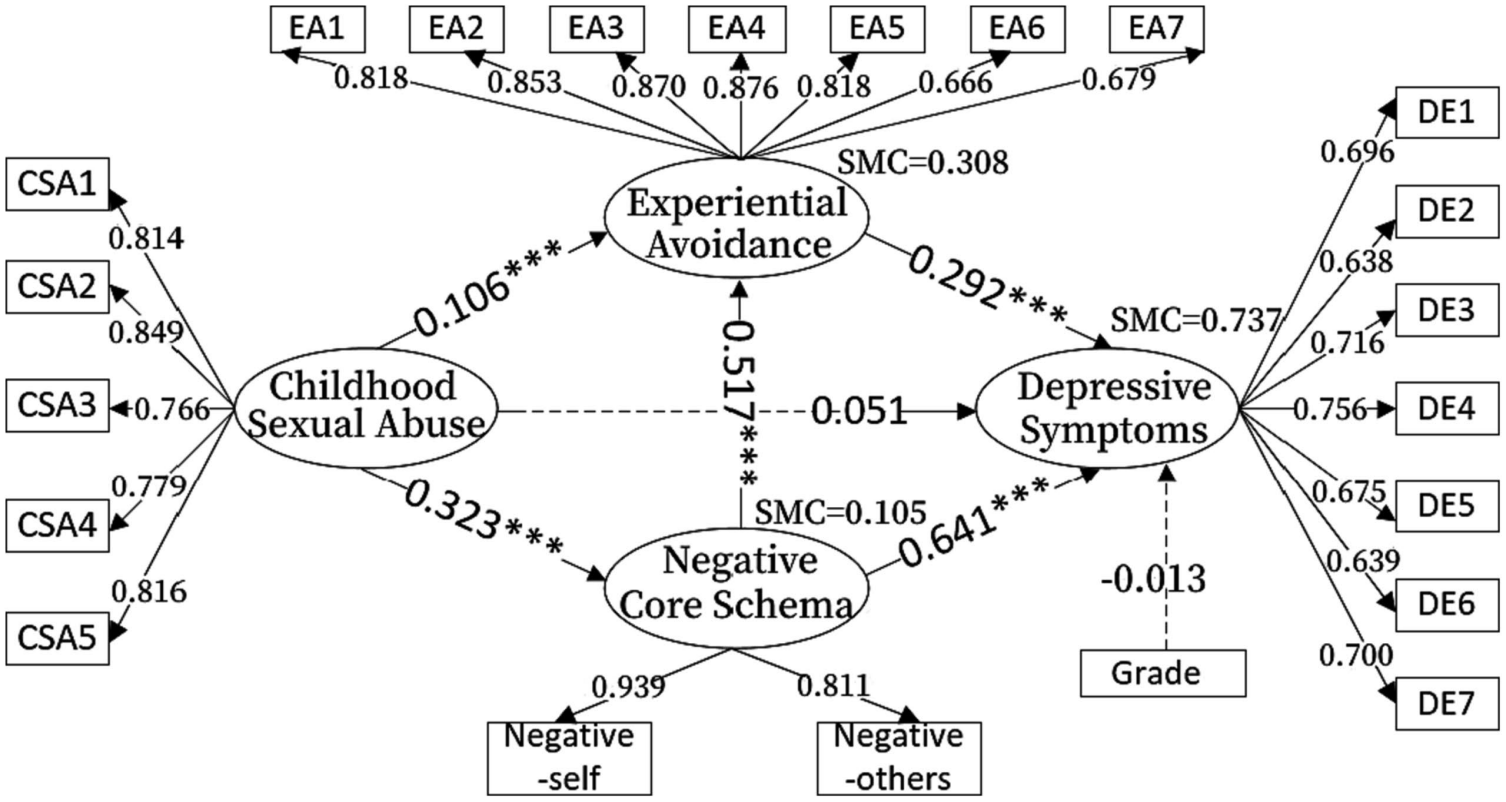
Serial mediation model with standardized estimates; ***p < 0.001.

Amos 23.0 was used to test the serial mediation model. Previous studies have shown that there is grade differences in depressive symptoms among college students (Liu and Wang, 2022), so grade was used as a control variable in this study. We used the comparative fit index (CFI), Tuck-Lewis index (TLI), and root mean square error of approximation (RMSEA) to evaluate the fit of the model. When CFI and TLI scores are above 0.90, and RMSEA value is less than 0.08, the model fit is adequate (Hu and Bentler, 1999). The results showed that the model fit is adequate (χ^2^/*df* = 3.422, RESEA = 0.069, CFI = 0.929, TLI = 0.919). The significance of each mediating effect was tested using the non-parametric bootstrap program, which was repeated 5,000 times to calculate the 95% confidence interval (CI). If the 95% CI did not include zero, the mediation effect was significant. The results showed that CSA did not directly predict depressive symptoms in female college students (β = 0.051, *p* = 0.139). The mediating effect of negative core schema was significant (β = 0.207, *p* < 0.01, 95CI = [0.265, 0.525]), with a relative mediating effect of 61.24%. The mediating effect of experiential avoidance was also significant (β = 0.031, *p* < 0.01, 95CI = [0.015, 0.118]), with a relative mediating effect of 9.17%. The serial mediating effect of CSA and experiential avoidance was significant as well (β = 0.049, *p* < 0.01, 95CI = [0.055, 0.136]), with a relative mediating effect of 14.50%. The effect values of the specific paths are shown in Table 2 and Figure 1.

**Table 2:** Mediating effects of negative core schema and experiential avoidance between CSA and depressive symptoms.

	Standardized effect	Unstandardized effect	Boot *SE*	Boot LLCI	Boot ULCI	Relative effect
Direct effect	0.051	0.091	0.061	−0.021	0.233	15.09%
CSA → NCS → DS	0.207	0.370	0.063	0.265	0.525	61.24%
CSA → EA → DS	0.031	0.055	0.025	0.015	0.118	9.17%
CSA → NCS → EA → DS	0.049	0.087	0.019	0.055	0.136	14.50%
Total indirect effect	0.287	0.513	0.075	0.383	0.674	
Total effect	0.338	0.604	0.090	0.429	0.792	

CSA, childhood sexual abuse; NCS, negative core schema; EA, experiential avoidance; DS, depressive symptoms. If the LLCI and ULCI do not include zero, the effect is considered statistically significant.

## Discussion

This study explored the relationship between CSA and negative core schema, experiential avoidance, and depressive symptoms. The results confirmed our hypotheses and revealed that negative core schema and experiential avoidance play a serial mediating role in the relationship between CSA and depressive symptoms in female college students. The results show that CSA is significantly positively correlated with depressive symptoms. This is consistent with previous studies which have found that adolescents who experience traumatic events are more likely to develop mental health problems (Keiley et al., 2001; Brown et al., 2016; Li et al., 2023). CSA was significantly positively correlated with depressive symptoms in college students (Zhang et al., 2022), and CSA had a positive predictive effect on depressive symptoms in female adolescents and adults (Trickett et al., 2011; Girard et al., 2021). According to the diathesis-stress model, CSA, as a distal stress event, will have a negative impact on individuals’ mental health (Broerman, 2020). Meanwhile, stress-oriented theory suggests that the accumulation of traumatic events such as CSA will cause the mental health problems such as depression in adolescents (Lazarus and Folkman, 1986).

Our results show that negative core schema plays a mediating role between CSA experiences and depressive symptoms (Plays the strongest mediating role in SEM), consistent with H1. This is in line with previous studies that have shown that negative schema play a mediating role between child mistreatment and adult depression (Cukor and McGinn, 2006; Cui et al., 2011; Rezaei and Ghazanfari, 2016). According CBT, childhood trauma such as CSA will cause individuals to form negative core schema, which affect individual information processing and thereby lead to emotional disorders (Beck et al., 1979). In other words, children and adolescents who have experienced sexual abuse are prone to feelings of insecurity and hostility, as well as other threatening factors in their surrounding environment. Because of this, their basic needs of safety, belongingness, and love are often not met, which leads them to gradually form negative schema that they as individuals are worthless, incapable, unworthy of love, and easily rejected by others. These negative schema, as models through which external information is processed, affect the individuals’ emotional responses to life events (Dozois et al., 2009), thus increasing their risk of mental health problems such as depression.

The results of the current study also suggest that experiential avoidance plays a mediating role between CSA and depressive symptoms, consistent with H2. This is consistent with the findings of previous studies, which have shown that experiential avoidance plays a mediating role between childhood trauma both externalizing problems (Roche et al., 2019) and depressive symptoms (Ghazanfari et al., 2018). Children and adolescents often have difficulty coping with traumatic events such as neglect or abuse on their own. They tend to cope by avoiding thoughts, emotions, body sensations, memories, and behaviors related to the trauma, which leads them to gradually acquire coping strategies of experiential avoidance. Meanwhile, according to ACT, childhood traumatic experiences can cause individuals to form unhelpful relational frameworks, which will cause individuals to adhere rigidly to these relational frames and adopt experiential avoidance to deal with them, thus exacerbating mental health problems such as depression and anxiety (Hayes et al., 2011). For example, CSA experiences cause female individuals to form relational frame conclusions such as, “I am flawed” and “I am not lovable,” making these individuals prone to producing a series of negative thought patterns and emotional experiences when faced with stressful life events, even causing memories or pictures related to their CSA to pop up in their minds. These cause the individual pain, which can be quickly stopped through experiential avoidance. Under such circumstances, individuals tend to use experiential avoidance more frequently to cope, thus adopting it as a coping strategy (Folkman et al., 1986; Roche et al., 2019). Overuse of experiential avoidance makes the reaction of avoidance occur more quickly and more frequently, however, and the pain experienced becomes more intense and serious than before, leading to mental health problems such as depression and anxiety (Hayes et al., 2006, 2011).

Finally, this study found that negative core schema can positively predict experiential avoidance, and negative core schema and experiential avoidance play a serial mediating role between CSA and depressive symptoms in female college students, thus validating H3. CBT theory holds that the cognitive triangle of feeling–thinking–action plays an important role in the formation and maintenance of mental health problems (Southam-Gerow et al., 1997). As a type of cognitive schema, negative core schema will cause information processing bias, produce negative thoughts and feelings, and encourage the adoption of adverse coping behaviors such as experiential avoidance to deal with the symptoms of depression and other mental disorders, thus maintaining and aggravating the mental health problems. According to ACT theory, relational frames play an important role in the formation of experiential avoidance (Hayes et al., 2011). In fact, relational frames and negative core schema are conceptually similar, and both are formed in the childhood environment. For example, a traumatic childhood experience causes an individual to form a negative core schema of “I am flawed” and “I am not lovable,” which ACT refers to as a negative relationship framing conclusion. Under the influence of such a negative core schema (or relational frame conclusion), individuals tend to view external stimuli as threats, and are prone to a series of automatic thoughts such as “I am not good enough” and “Others make fun of me,” resulting in emotional distress, causing or exacerbating the symptoms of depression. To avoid these negative experiences as much as possible, individuals will adopt experiential avoidance (e.g., avoiding social scenes, or avoiding certain thoughts, memories, and feelings), but in the long run, this will maintain or even aggravate the symptoms of anxiety and depression.

## Implications

This study considered the mechanism of negative core schema and experiential avoidance in the influence of CSA experiences on female college students’ depressive symptoms, and provides theoretical guidance for the prevention of female college students’ depression. In college mental health education, special attention should be paid to female college students who have suffered CSA experiences, because the negative schema formed by these individuals in their growth process makes them more susceptible to the development of depressive symptoms. Meanwhile, awareness and understanding of experiential avoidance and acceptance should be taught in mental health education in colleges and universities, to help female college students learn to recognize experiential avoidance and use acceptance to deal with the thoughts, emotions, physical sensations, and memories that trouble them.

## Limitations and future research

There are some shortcomings to this study. First, due to the need to recall CSA through a self-report method, we cannot rule out the possibility that study participants had memory problems or were unwilling to mention past adverse situations. As self-reporting can have problems with subjectivity and reliability, future research should collect data from multiple sources, such as parents, teachers, or peers. Second, this study is a cross-sectional study, which limits the ways in which the data can be interpreted. It only shows correlations between variables, not causation. Future research should test the present mediational model longitudinally in a multi-wave prospective study to allow for testing of the hypothesized unfolding of the sequential mediating effects over time. Third, this study did not control for the overlap of CSA with physical abuse, emotional abuse, or neglect which should be assessed and controlled in future to establish specificity to CSA. Fourth, limited by space, only two mediating variables, negative core schema and empirical avoidance, are considered in this study. In fact, there are other possible mediating variables worth considering, such as domestic violence (DiLillo et al., 2001). Therefore, future studies should further investigate the mediating roles of variables such as domestic violence. Fifth, the sample in this study included students from only two universities in mainland China, limiting the generalizability of our findings. Future studies should verify the conclusions of this study using a larger sample. Sixth, this study only considered the mechanism of CSA in terms of individual psychological factors, however the roles of genetic and biological factors (such as oxytocin receptor genes, Olazábal et al., 2023) should be considered in the future.

## Data availability statement

The raw data supporting the conclusions of this article will be made available by the authors, without undue reservation.

## Ethics statement

The studies involving humans were approved by the Ethics Committee of Hunan Normal University, Hunan Province, China. The studies were conducted in accordance with the local legislation and institutional requirements. The participants provided their written informed consent to participate in this study.

## Author contributions

HZ: Conceptualization, Investigation, Project administration, Writing – original draft. YC: Conceptualization, Validation, Writing – original draft. LL: Conceptualization, Formal analysis, Funding acquisition, Project administration, Resources, Supervision, Validation, Writing – review & editing. BP: Conceptualization, Data curation, Methodology, Software, Writing – review & editing.

## References

[ref1] Akbari M. Seydavi M. Hosseini Z. S. Krafft J. Levin M. E. (2022). Experiential avoidance in depression, anxiety, obsessive-compulsive related, and posttraumatic stress disorders: a comprehensive systematic review and meta-analysis. J. Context. Behav. Sci. 24, 65–78. doi: 10.1016/j.jcbs.2022.03.007

[ref2] Angelakis I. Gooding P. (2021). Experiential avoidance in non-suicidal self-injury and suicide experiences: a systematic review and meta-analysis. Suicide Life-Threat. Behav. 51, 978–992. doi: 10.1111/sltb.12784, PMID: 34184775

[ref3] Appiah-Kusi E. Fisher H. L. Petros N. Wilson R. Mondelli V. Garety P. A.. (2017). Do cognitive schema mediate the association between childhood trauma and being at ultra-high risk for psychosis? J. Psychiat. Res. 88, 89–96. doi: 10.1016/j.jpsychires.2017.01.003, PMID: 28103519

[ref4] Batten S. V. Follette V. M. Aban I. B. (2002). Experiential avoidance and high-risk sexual behavior in survivors of child sexual abuse. J. Child Sex. Abus. 10, 101–120. doi: 10.1300/J070v10n02_06, PMID: 15154403

[ref5] Beck A. T. (1987). “Cognitive approaches to panic disorder: theory and therapy” in Panic: Psychological perspectives. eds. Rachman S. Master J. (London: Routledge), 91–109.

[ref6] Beck A. T. (1991). Cognitive therapy. A 30-year retrospective. Am. Psychol. 46, 368–375. doi: 10.1037//0003-066x.46.4.368, PMID: 2048795

[ref7] Beck A. T. (1999). “Cognitive aspects of personality disorders and their relation to syndromal disorders: a psychoevolutionary approach” in Personality and psychopathology. ed. Cloninger C. R. (Washington: American Psychiatric Press), 411–429.

[ref8] Beck J. S. (2011). Cognitive behavior therapy: Basics and beyond (2nd). New York: Guilford Press.

[ref9] Beck A. T. Rush A. J. Shaw B. F. Emery G. (1979). Cognitive therapy of depression. New York: Guilford Press.

[ref10] Bernstein D. P. Ahluvalia T. Pogge D. Handelsman L. (1997). Validity of the childhood trauma questionnaire in an adolescent psychiatric population. J. Am. Acad. Child Adoles. Psychiat. 36, 340–348. doi: 10.1097/00004583-199703000-00012 9055514

[ref11] Bishop A. Younan R. Low J. Pilkington P. D. (2022). Early maladaptive schemas and depression in adulthood: a systematic review and meta-analysis. Clin. Psychol. Psychot. 29, 111–130. doi: 10.1002/cpp.2630, PMID: 34131990

[ref12] Bond F. W. Hayes S. C. Baer R. A. Carpenter K. M. Guenole N. Orcutt H. K.. (2011). Preliminary psychometric properties of the acceptance and action questionnaire-II: a revised measure of psychological inflexibility and experiential avoidance. Behav. Ther. 42, 676–688. doi: 10.1016/j.beth.2011.03.007, PMID: 22035996

[ref13] Bradley B. Mathews A. (1983). Negative self-schemata in clinical depression. Brit. J. Clin. Psychol. 22, 173–181. doi: 10.1111/j.2044-8260.1983.tb00598.x 6626790

[ref14] Broerman R. (2020). “Diathesis-stress model” in Encyclopedia of personality and individual differences. eds. Zeigler-Hill V. Shackelford T. K. (Berlin: Springer), 1107–1109.

[ref15] Brown S. Fite P. J. Stone K. Bortolato M. (2016). Accounting for the associations between child maltreatment and internalizing problems: the role of alexithymia. Child Abuse Negl. 52, 20–28. doi: 10.1016/j.chiabu.2015.12.008, PMID: 26774529 PMC12849041

[ref16] Cao J. Ji Y. Zhu Z. (2013). Reliability and validity of the Chinese version of the acceptance and action questionnaire-second edition (AAQ-II) in college students. Chin. Ment. Health J. 27, 873–877. doi: 10.3969/j.issn.1000-6729.2013.11.014

[ref17] Chen J. Liu Y. Tan Q. (2022). The relationship of cumulative ecological risk and higher vocational college Students'Learning burnout:the mediation effect of negative self-schema and internet addiction. Psychol. Dev. Edu. 38, 576–583. doi: 10.16187/j.cnki.issn1001-4918.2022.04.14

[ref18] Cui L. X. Luo X. J. Xiao J. (2011). The influence of childhood trauma on trait-depression and trait-anxiety: the mediation-specificity of schemas. Acta Psychol. Sin. 43, 1163–1174. doi: 10.3724/SP.J.1041.2011.01163

[ref19] Cukor D. McGinn L. K. (2006). History of child abuse and severity of adult depression: the mediating role of cognitive schema. J. Child Sex. Abus. 15, 19–34. doi: 10.1300/J070v15n03_02 16893817

[ref20] DiLillo D. Giuffre D. Tremblay G. C. Peterson L. (2001). A closer look at the nature of intimate partner violence reported by women with a history of child sexual abuse. J. Interpers. Violence 16, 116–132. doi: 10.1177/088626001016002002

[ref21] Dionne F. Ngô T. L. Blais M. C. (2013). The psychological flexibility model: a new approach to mental health. Sante Ment. Que. 38, 111–130. doi: 10.7202/1023992ar, PMID: 24719005

[ref22] Dozois D. J. A. Beck A. T. (2008) in “Cognitive schemas, beliefs and assumptions” in risk factors in depression. ed. Dobson K. S. (Amsterdam: Elsevier Academic Press), 119–143.

[ref23] Dozois D. J. Martin R. A. Bieling P. J. (2009). Early maladaptive schemas and adaptive/maladaptive styles of humor. Cognitive Ther. Res. 33, 585–596. doi: 10.1007/s10608-008-9223-9

[ref24] Evans J. Heron J. Lewis G. Araya R. Wolke D. (2005). Negative self-schemas and the onset of depression in women: longitudinal study. Brit. J. Psychiat. 186, 302–307. doi: 10.1192/bjp.186.4.302 15802686

[ref25] Fergusson D. M. McLeod G. F. Horwood L. J. (2013). Childhood sexual abuse and adult developmental outcomes: findings from a 30-year longitudinal study in New Zealand. Child Abuse Negl. 37, 664–674. doi: 10.1016/j.chiabu.2013.03.013, PMID: 23623446

[ref26] Folkman S. Lazarus R. S. Gruen R. J. DeLongis A. (1986). Appraisal, coping, health status, and psychological symptoms. J. Pers. Soc. Psychol. 50, 571–579. doi: 10.1037/0022-3514.50.3.571 3701593

[ref27] Fowler D. Freeman D. Smith B. E. N. Kuipers E. Bebbington P. Bashforth H.. (2006). The brief Core Schema scales (BCSS): psychometric properties and associations with paranoia and grandiosity in non-clinical and psychosis samples. Psychol. Med. 36, 749–759. doi: 10.1017/S0033291706007355, PMID: 16563204

[ref28] Fu X. L. Zhang K. (2023). Report on National Mental Health Development in China 2021–2022. Beijing: Social Sciences Academic Press.

[ref29] Ghazanfari F. Rezaei M. Rezaei F. (2018). The mediating role of repetitive negative thinking and experiential avoidance on the relationship between childhood trauma and depression. Arch. Psychiat. Nurs. 32, 432–438. doi: 10.1016/j.apnu.2017.12.010, PMID: 29784226

[ref30] Girard M. Hébert M. Godbout N. Cyr M. Frappier J. Y. (2021). A longitudinal study of suicidal ideation in sexually abused adolescent girls: depressive symptoms and affect dysregulation as predictors. J. Trauma. Stress. 34, 1132–1138. doi: 10.1002/jts.22608, PMID: 33078516

[ref31] Gong X. Xie X. Xu R. Luo Y. (2010). Psychometric properties of the Chinese versions of DASS-21 in Chinese college students. Chin. J. Clin. Psychol. 18, 443–446. doi: 10.16128/j.cnki.1005-3611.2010.04.020

[ref32] Harris R. (2019). ACT made simple: An easy-to-read primer on acceptance and commitment therapy (2nd). Oakland: New Harbinger Publications.

[ref33] Hayes S. C. Luoma J. B. Bond F. W. Masuda A. Lillis J. (2006). Acceptance and commitment therapy: model, processes and outcomes. Behav. Res. Ther. 44, 1–25. doi: 10.1016/J.BRAT.2005.06.006 16300724

[ref34] Hayes S. C. Strosahl K. D. Wilson K. G. (2011). Acceptance and commitment therapy: The process and practice of mindful change. New York: Guilford Press.

[ref35] Hayes S. C. Wilson K. G. Gifford E. V. Follette V. M. Strosahl K. (1996). Experiential avoidance and behavioral disorders: a functional dimensional approach to diagnosis and treatment. J. Consult. Clin. Psychol. 64, 1152–1168. doi: 10.1037/0022-006x.64.6.1152, PMID: 8991302

[ref36] Hébert M. Smith K. Caouette J. Cénat J. M. Karray A. Cartierre N.. (2021). Prevalence and associated mental health outcomes of child sexual abuse in youth in France: observations from a convenience sample. J. Affect. Disorders 282, 820–828. doi: 10.1016/j.jad.2020.12.100, PMID: 33601723

[ref37] Heshmati R. Azmoodeh S. Caltabiano M. L. (2021). Pathway linking different types of childhood trauma to somatic symptoms in a subclinical sample of female college students: the mediating role of experiential avoidance. J. Nerv. Ment. Dis. 209, 497–504. doi: 10.1097/NMD.0000000000001323, PMID: 34170858

[ref38] Hillberg T. Hamilton-Giachritsis C. Dixon L. (2011). Review of meta-analyses on the association between child sexual abuse and adult mental health difficulties: a systematic approach. Trauma Violence Abuse 12, 38–49. doi: 10.1177/15248380103868, PMID: 21288934

[ref39] Hu L. Bentler P. M. (1999). Cutoff criteria for fit indexes in covariance structure analysis: conventional criteria versus new alternatives. Struct. Equ. Model. 6, 1–55. doi: 10.1080/10705519909540118

[ref40] Keiley M. K. Howe T. R. Dodge K. A. Bates J. E. Pettit G. S. (2001). The timing of child physical maltreatment: a cross-domain growth analysis of impact on adolescent externalizing and internalizing problems. Dev. Psychopathol. 13, 891–912. doi: 10.1017/S0954579401004084, PMID: 11771913 PMC2769082

[ref41] Kessler R. C. (2003). Epidemiology of women and depression. J. Affect. Disorders 74, 5–13. doi: 10.1016/S0165-0327(02)00426-3 12646294

[ref42] Kheirabadi H. Jajarmi M. Bakhshipoor A. (2021). A structural correlation modeling of stress and substance abuse with the mediating role of meaning in life and experiential avoidance. Int. J. Behav. Sci. 15, 34–40. doi: 10.30491/IJBS.2021.225787.1241

[ref43] Lazarus R. S. Folkman S. (1986). ““Cognitive theories of stress and the issue of circularity” in dynamics of stress: physiological, psychological, and social perspectives” in The plenum series on stress and coping. eds. Appley M. H. Trumbull R. (New York: Plenum Press), 63–80.

[ref44] Leeb R. T. (2008). Child maltreatment surveillance: Uniform definitions for public health and recommended data elements. Atlanta Centers for Disease Control and Prevention, National Center for Injury Prevention and Control.

[ref45] Li E. T. Luyten P. Midgley N. (2020). Psychological mediators of the association between childhood emotional abuse and depression: a systematic review. Front. Psych. 11:559213. doi: 10.3389/fpsyt.2020.559213, PMID: 33343409 PMC7746653

[ref46] Li A. Wang S. Liu X. (2023). Childhood psychological maltreatment and life satisfaction among Chinese young adults: the mediating role of internalizing problems and the buffering role of social support. Curr. Psychol. 42, 7701–7711. doi: 10.1007/s12144-021-02126-3

[ref47] Litwin R. Goldbacher E. M. Cardaciotto L. Gambrel L. E. (2017). Negative emotions and emotional eating: the mediating role of experiential avoidance. Eat. Weight Disord-St. 22, 97–104. doi: 10.1007/s40519-016-0301-9, PMID: 27460010

[ref48] Liu A. Wang R. (2022). Latent profile analysis on the characteristics of college students’ depression. Chin. J. Clin. Psychol. 30, 1208–1212. doi: 10.16128/j.cnki.1005-3611.2022.05.040

[ref49] Liu H. Wang W. Yang J. Guo F. Yin Z. (2021). The effects of alexithymia, experiential avoidance, and childhood sexual abuse on non-suicidal self-injury and suicidal ideation among Chinese college students with a history of childhood sexual abuse. J. Affect. Disorders 282, 272–279. doi: 10.1016/j.jad.2020.12.181, PMID: 33418378

[ref440] Lovibond S. H. (1995). Manual for the depression anxiety stress scales (2nd). Sydney: University of New South Wales.

[ref901] Lovibond P. F. Lovibond S. H. (1995). The structure of negative emotional states: comparison of the Depression Anxiety Stress Scales (DASS) with the beck depression and anxiety inventories. Behav. Res. Ther. 33, 335–343. 10.1016/0005-7967(94)00075-u 7726811

[ref51] Maniglio R. (2010). Child sexual abuse in the etiology of depression: a systematic review of reviews. Depress. Anxiety 27, 631–642. doi: 10.1002/da.20687, PMID: 20336807

[ref52] May T. Younan R. Pilkington P. D. (2022). Adolescent maladaptive schemas and childhood abuse and neglect: a systematic review and meta-analysis. Clin. Psychol. Psychot. 29, 1159–1171. doi: 10.1002/cpp.2712, PMID: 35060262 PMC9544896

[ref53] Murray L. K. Nguyen A. Cohen J. A. (2014). Child sexual abuse. Child Adolesc. Psychiat. Clin. 23, 321–337. doi: 10.1016/j.chc.2014.01.003, PMID: 24656583 PMC4413451

[ref54] Noble R. E. (2005). Depression in women. Metabolism 54, 49–52. doi: 10.1016/j.metabol.2005.01.014 15877314

[ref55] Nunnally J. C. Bernstein I. H. (1994). Psychometric theory. 3rd. New York: McGraw -Hill.

[ref56] Olazábal D. E. Bertoni B. Grandi G. Musetti D. Rey G. Sandberg N.. (2023). Oxytocin system polymorphisms rs237887 and rs2740210 variants increase the risk of depression in pregnant women with early abuse. Dev. Psychobiol. 65:e22400. doi: 10.1002/dev.22400, PMID: 37338248

[ref57] Orcutt H. K. Reffi A. N. Ellis R. A. (2020). “Experiential avoidance and PTSD” in Emotion in posttraumatic stress disorder: Etiology, assessment, neurobiology, and treatment. eds. Tull M. T. Kimbrel N. A. (Amsterdam: Elsevier Academic Press), 409–436.

[ref58] Peng B. Hu N. Yu H. Xiao H. Luo J. (2021). Parenting style and adolescent mental health: the chain mediating effects of self-esteem and psychological inflexibility. Front. Psychol. 12:738170. doi: 10.3389/fpsyg.2021.738170, PMID: 34721210 PMC8548717

[ref59] Podsakoff P. M. MacKenzie S. B. Lee J. Y. Podsakoff N. P. (2003). Common method biases in behavioral research: a critical review of the literature and recommended remedies. J. Appl. Psychol. 88, 879–903. doi: 10.1037/0021-9010.88.5.879, PMID: 14516251

[ref60] Polusny M. A. Rosenthal M. Z. Aban I. Follette V. M. (2004). Experimental avoidance as a mediator of the effects of adolescent sexual victimization on negative adult outcomes. Violence Victims 19, 109–120. doi: 10.1891/vivi.19.1.109.33238, PMID: 15179750

[ref61] Rellini A. H. Meston C. M. (2011). Sexual self-schemas, sexual dysfunction, and the sexual responses of women with a history of childhood sexual abuse. Arch. Sex. Behav. 40, 351–362. doi: 10.1007/s10508-010-9694-0, PMID: 21140286 PMC3047701

[ref62] Rezaei M. Ghazanfari F. (2016). The role of childhood trauma, early maladaptive schemas, emotional schemas and experimental avoidance on depression: a structural equation modeling. Psychiatry Res. 246, 407–414. doi: 10.1016/j.psychres.2016.10.037, PMID: 27788461

[ref63] Roche A. I. Kroska E. B. Miller M. L. Kroska S. K. O'Hara M. W. (2019). Childhood trauma and problem behavior: examining the mediating roles of experiential avoidance and mindfulness processes. J. Am. Coll. Heal. 67, 17–26. doi: 10.1080/07448481.2018.1455689, PMID: 29565779 PMC6296903

[ref64] Russell D. Higgins D. Posso A. (2020). Preventing child sexual abuse: a systematic review of interventions and their efficacy in developing countries. Child Abuse Negl. 102:104395. doi: 10.1016/j.chiabu.2020.104395, PMID: 32062425

[ref65] Salk R. H. Hyde J. S. Abramson L. Y. (2017). Gender differences in depression in representative national samples: Meta-analyses of diagnoses and symptoms. Psych. Bull. 143, 783–822. doi: 10.1037/bul0000102, PMID: 28447828 PMC5532074

[ref66] Southam-Gerow M. A. Henin A. Chu B. Marrs A. Kendall P. C. (1997). Cognitive-behavioral therapy with children and adolescents. Child Adol. Psych. Clin. 6, 111–136. doi: 10.1016/s1056-4993(18)30323-7

[ref67] Trickett P. K. Noll J. G. Putnam F. W. (2011). The impact of sexual abuse on female development: lessons from a multigenerational, longitudinal research study. Dev. Psychopathol. 23, 453–476. doi: 10.1017/S0954579411000174, PMID: 23786689 PMC3693773

[ref68] Tull M. T. Gratz K. L. Salters K. Roemer L. (2004). The role of experiential avoidance in posttraumatic stress symptoms and symptoms of depression, anxiety, and somatization. J. Nerv. Ment. Dis. 192, 754–761. doi: 10.1097/01.nmd.0000144694.30121.89, PMID: 15505519

[ref69] Wang M. Y. Han F. F. Liu J. Huang K. L. Huang K. L. Peng H. Y.. (2020). A meta-analysis of detection rate of depression symptoms and related factors in college students. Chin. Ment. Health J. 34, 1041–1047. doi: 10.3969/j.issn.1000-6729.2020.12.012

[ref70] Weiss E. L. Longhurst J. G. Mazure C. M. (1999). Childhood sexual abuse as a risk factor for depression in women: psychosocial and neurobiological correlates. Am. J. Psychiatry 156, 816–828. doi: 10.1176/ajp.156.6.816, PMID: 10360118

[ref71] Whiffen V. E. MacIntosh H. B. (2005). Mediators of the link between childhood sexual abuse and emotional distress: a critical review. Trauma Violence Abuse 6, 24–39. doi: 10.1177/1524838004272543, PMID: 15574671

[ref72] Wilson L. C. Scarpa A. (2015). Interpersonal difficulties mediate the relationship between child sexual abuse and depression symptoms. Violence Victims. 30, 163–176. doi: 10.1891/0886-6708.VV-D-13-00059, PMID: 25774421

[ref73] World Health Organization (2023). Depression. Available at: https://www.who.int/health-topics/depression#tab=tab_1

[ref74] Yaroslavsky I. Bush A. H. France C. M. (2022). Emotion regulation deficits mediate childhood sexual abuse effects on stress sensitization and depression outcomes. Dev. Psychopathol. 34, 157–170. doi: 10.1017/S095457942000098X 33023709

[ref75] Young J. E. Klosko J. S. Weishaar M. E. (2003). Schema therapy: A practitioner’s guide. New York: Guilford Press.

[ref76] Zhang R. Liang Y. Cao W. Zeng L. Tang K. (2022). Sex and urban-rural differences in the relationship between childhood sexual abuse and mental health among Chinese college students. Int. J. Env. Res. Pub. He. 19:9225. doi: 10.3390/ijerph19159225, PMID: 35954586 PMC9368484

[ref77] Zhao X. Zhang Y. Li L. F. Zhou Y. Li H. Yang S. (2005). Reliability and validity of the Chinese version of childhood. Chin. J. Clin. Rehabil. 9, 105–107. doi: 10.3321/j.issn:1673-8225.2005.20.052

[ref78] Zou T. Yao S. Q. (2006). Cognitive vulnerability-stress model of depression: origin, development and integration. Adv. Psychol. Sci. 14, 762–768. doi: 10.3969/j.issn.1671-3710.2006.05.018

